# Impact of Implementation of BacT*/*Alert Virtuo on Blood Culture Time to Positivity in Sepsis Patients

**DOI:** 10.1128/spectrum.05003-22

**Published:** 2023-02-07

**Authors:** Tony Mazzulli, Dimitrije Ratkov, Kristyn Guglielmin, Bharat Gandhi, Richard Remington, Robert Birch, Louise Zimmer, Cherilyn D. Garner

**Affiliations:** a Department of Microbiology, University Health Network/Mount Sinai Hospital, Toronto, Ontario, Canada; b Department of Laboratory Medicine and Pathobiology, University of Toronto, Toronto, Ontario, Canada; c bioMérieux, Inc., Hazelwood, Missouri, USA; d bioMérieux, Inc., Durham, North Carolina, USA; University of Maryland School of Medicine

**Keywords:** Virtuo, blood cultures, sepsis, time to positivity

## Abstract

Time to positivity (TTP) for blood culture bottles incubated in the BacT/Alert Virtuo instrument was compared to the BacT/Alert 3D. TTP was significantly shorter with the Virtuo (median 16.2 h) than 3D (median 21.1 h; *P < *0.001). Switching from 3D to Virtuo significantly improved TTP in this multicenter hospital setting study.

**IMPORTANCE** Sepsis affects millions of people around the world each year, and accounts for a significant number of deaths in hospital intensive care units (ICU). Timely diagnosis is key to decreasing morbidity and mortality. One important element of sepsis diagnosis is organism detection using blood cultures. In this study, we examined the impact of implementing the BacT/Alert Virtuo automated blood culture detection system on time to positivity in an ICU patient population at a multicenter hospital setting.

## OBSERVATION

Sepsis affects millions of people around the world each year, and accounts for nearly one quarter of all deaths in hospital intensive care units (1). Timely diagnosis is key to decreasing morbidity and mortality (2). One important element of sepsis diagnosis is organism detection using blood cultures. Historically, blood cultures were manual and time-consuming; but with the advent of automated monitoring instruments, time to positivity has decreased. BacT/Alert Virtuo, a more advanced version of automated monitoring instrument, has been shown to have lower TTP than other current instruments (3–8). In this study, we examined the impact of implementing the Virtuo instrument on TTP in an ICU patient population at a multicenter hospital setting.

This study was a retrospective preimplementation/postimplementation study that included adult patients (≥18 years of age) admitted to a medical-surgical ICU at one of three Toronto, Canada hospitals (Mount Sinai Hospital, Toronto General Hospital, or Toronto Western Hospital) with suspected sepsis (bacteremia or fungemia) and an index positive blood culture. Only the first positive blood culture for each patient was included. The study was reviewed by the Mount Sinai Research Ethics Review Board, and it was determined that informed consent was not required. Samples for the BacT/Alert 3D were collected between February 2016 and March 2017 and samples for the BacT/Alert Virtuo were collected between May 2017and April 2018. BacT/Alert FA Plus and FN Plus bottles were used at all hospitals for both pre and post study arms.

The primary objective was to compare the TTP from blood culture bottles with the 3D and Virtuo systems. TTP was measured as the length of time from when a blood culture bottle entered the incubator to the time the culture signaled positive. Workflow endpoints were analyzed overall as well as by Gram stain result. Median and IQR were calculated for workflow endpoints for the 3D and Virtuo patient groups. Differences between 3D and Virtuo performance were tested with independent 2-group Mann-Whitney U Test for total isolates, for the subset of isolates where further antimicrobial susceptibility testing (AST) was performed, and for the subsets based on Gram stain. The subset of isolates with AST was analyzed separately as it was considered more likely to contain clinically significant isolates, since AST is less likely to be performed for isolates classified as contaminants. The R statistical language (version 4.1.2) was used for statistical analysis (9).

There were 115 positive blood cultures tested on the 3D system and 114 positive blood cultures tested on the Virtuo system. For both Virtuo and 3D, the most common Gram-positive organisms isolated were staphylococci other than Staphylococcus aureus (Table 1) and the most common Gram-negative organism was Escherichia coli (Table 1). The median TTP was 21.1 h with the 3D system and 16.2 h with the Virtuo system, including fungal positives (*P < *0.001), and 21.1 and 15.5 h, respectively, when fungal positives were excluded (*P < *0.001) (Table 2). Among patient isolates where AST was performed, median TTP was 16.0 h with the 3D system and 11.1 h with the Virtuo system, including 4 fungal positives (*P = *0.008) and 16.0 h and 10.9 h, respectively, when fungal positives were excluded (*P < *0.001) (Table 2).

**TABLE 1:** Number of species isolated from blood cultures

Organism	Total	3D	Virtuo
Breakdown of Gram positive microorganisms			
Bacteria			
Staphylococci other than Staphylococcus aureus	88	41	47
Staphylococcus aureus	26	16	10
*Bacillus* spp. not B. anthracis	11	6	5
Viridans group streptococci	10	5	5
Cutibacterium acnes	4	2	2
Streptococcus pneumoniae	3	1	2
*Propionibacterium* spp.	3	3	0
Enterococcus faecalis	3	1	2
Streptococcus anginosus group	2	1	1
*Gemella* spp.	2	2	0
Enterococcus faecium	2	1	1
*Corynebacterium* spp.	2	0	2
Streptococcus pyogenes	2	1	1
Streptococcus intermedius	1	0	1
Staphylococcus pseudintermedius	1	0	1
*Peptostreptococcus* spp.	1	1	0
*Paenibacillus* spp.	2	2	0
*Micrococcus* spp.	1	0	1
Enterococcus avium	1	0	1
Dermabacter hominis	1	0	1
*Brevibacillus* spp.	2	1	1
*Abiotrophia* spp.	1	0	1
Yeast			
Candida albicans	2	0	2
Candida glabrata	2	0	2
Candida parapsilosis	1	0	1
Candida tropicalis	1	0	1
	175	84	91
Breakdown of Gram-negative bacteria			
Escherichia coli	24	15	9
Enterobacter cloacae complex	6	2	4
Proteus mirabilis	5	4	1
Pseudomonas aeruginosa	5	2	3
Serratia marcescens	5	3	2
Klebsiella pneumoniae	4	2	2
Bacteroides fragilis group	2	2	0
Klebsiella aerogenes	1	0	1
*Prevotella* spp.	1	0	1
Salmonella enteritidis	1	0	1
Stenotrophomonas maltophilia	1	0	1
	55	30	25

**TABLE 2:** Blood culture time to positive results

	3D			Virtuo			
Time to positive (hours)	n	Median	IQR	n	Median	IQR	*P*
All samples	115	21.1	15.2, 26.0	114	16.2	10.5, 24.1	<0.001
All samples, omitting Fungal	115	21.1	15.2, 26.0	108	15.5	10.2, 22.2	<0.001
Patient samples with AST	49	16.0	13.4, 19.7	51	11.1	8.0, 19.1	0.008
Patients samples with AST omitting fungal	49	16.0	13.4, 19.7	47	10.9	7.8, 18.3	<0.001
All monomicrobial Gram-negative samples	29	17.1	13.4, 22.0	22	10.2	6.7, 13.9	<0.001
Monomicrobial Gram-negative patient samples with AST	22	15.5	13.3, 18.1	20	10.1	6.0, 12.4	<0.001
All monomicrobial Gram-positive bacterial samples	81	21.5	16.6, 26.1	80	17.2	12.1, 24.1	0.003
Monomicrobial Gram-positive patient bacterial samples with AST	25	18.6	15.1, 22.5	21	13.8	8.8, 19.2	0.189

When divided by Gram reaction, median TTP for Gram-negative bacteria was 17.1 h with the 3D system and 10.2 h with the Virtuo system (*P < *0.001) and among patient isolates where AST was performed, 15.5 h with the 3D system and 10.1 h with the Virtuo system (*P < *0.001) (Table 2). For Gram-positive bacteria, median TTP was 21.5 h with the 3D system and 17.2 h with the Virtuo system (*P = *0.003) (Table 2).

These results show that compared to 3D, Virtuo significantly improved TTP in ICU patients in this multicenter study. This was true for both Gram-negative and Gram-positive bacteria. This reduction in TTP for Virtuo was more marked than reported in previous clinical studies (4, 7). As the current study only examined TTP (time from culture bottle entering the incubator to the time the culture signaled positive), there were no differences in workflow or processing of blood cultures, training of staff, change in staff, or time between drawing of BC and loading bottles into the instrument that could explain the larger difference in TTP observed compared to previously published studies. However, this study focused on a specific ICU population, so it is possible the observed difference may be a result of the high acuity of this specific patient population, but additional studies would be necessary to confirm this impact. Shortened TTP has the potential to positively impact diagnosis and management of bloodstream infection-originated sepsis; however, further studies are needed to determine the clinical impact (e.g., antimicrobial usage, appropriateness of therapy, etc.).
